# Predicting undergraduate nursing students’ willingness to care for older adults: A multicenter cross‐sectional study in Asia

**DOI:** 10.1002/nop2.916

**Published:** 2021-05-06

**Authors:** Xian‐Liang Liu, Hui‐Lin Cheng, Simon Ching Lam

**Affiliations:** ^1^ College of Nursing and Midwifery Charles Darwin University Brisbane Qld Australia; ^2^ School of Nursing The Hong Kong Polytechnic University Hong Kong Hong Kong

**Keywords:** attitudes, demographics, older people, perception, predictor, willingness to care

## Abstract

**Aim:**

To investigate the willingness of nursing students in Hong Kong and Mainland China to care for the elderly and to identify the factors influencing their willingness.

**Design:**

A correlational and cross‐sectional design.

**Methods:**

A four‐part self‐administered questionnaire was utilized to collect data from the undergraduate nursing students in Hong Kong and Mainland China.

**Results:**

The total sample consisted of 820 nursing students, with 481 students in Hong Kong and 339 students in Mainland China. The scores of willingness to care for older people of the two groups were similar (mean score 4.68 versus 4.44), and no significant difference was observed between the students in the two regions (*p* = .071). A very low proportion of the nursing students in Hong Kong (7.7%) and those in Mainland China (3.6%) ranked caring for older people as their first choice of work. No salient predictors of willingness to care for older people existed for the nursing students in Mainland China.

## 1 INTRODUCTION

The world is getting older, particularly, Europe, North America, and Asia, which are experiencing a rapid increase in the number of older people (Li et al., 2019). The Chinese population is the fastest ageing population in the world, with people aged ≥65 years predicted to account for 17.2% to over 30% of the population of Mainland China, Hong Kong, Macau, and Taiwan in 2030 (He, Goodkind, & Kowal, 2016). China's ageing rate is expected to increase 13.24% points from 2019 to 2044 (Chen et al., 2019). Older people are generally the largest consumers of healthcare services, as ageing can lead to multiple health issues (Prince et al., 2015).

Universities are responsible for training sufficient numbers of nursing students to meet the demands of older people care and motivating them to care for older patients in various care settings (Chi et al., 2016). Nursing care skills are taught to nursing students to enable them to deliver healthcare services to older patients and their families effectively and efficiently (Patton & Henry, 2019). However, despite the teaching of core concepts in the nursing curriculum, the level of willingness to care for older people among undergraduate nursing students is low (Henderson et al., 2008; Rathnayake et al., 2016) or average (Che et al., 2018; Zhang et al., 2016). According to a longitudinal study, undergraduate nursing students’ willingness to care for older patients decreases from 8% (at the beginning of their course) to 2% (in their third year in the undergraduate nursing program), with only three out of 150 nursing students expressing their desire to work with older adults (Stevens, 2011).

## 2 BACKGROUND

Nursing students with a low level of willingness and negative attitude towards healthcare for older people can negatively affect the development or sustainability of healthcare services for the aged (Hanson, 2014). Furthermore, the reasons behind the willingness to care for older adults and other care‐related challenges among nursing students are often overlooked and thus not addressed (Chi et al., 2016). A challenge for future nurses is to develop their willingness to care for older patients as well as a positive attitude towards ageing (Xiao et al., 2013). Evaluating undergraduate nursing students’ attitude towards ageing and willingness to care for older patients to find a solution to this problem would be beneficial (King et al., 2013; Zhang et al., 2016).

Willingness to care for people is defined as a person's relative readiness and absence of reluctance to care for other individuals (Toygar et al., 2020; Zhao et al., 2020). The measurements for willingness to care for older people are diverse, including the willingness towards care scale (15 items; Chi et al., 2016; Liu, 2001), intent to work with older people scale (11 items; Che et al., 2018), ranking willingness to care for different age groups (King et al., 2013; Rathnayake et al., 2016), and self‐designed surveys using three‐option questions (Haron et al., 2013) or five‐point Likert scales (five items; Zhang et al., 2016). Ranking willingness to care for different age groups is the most commonly used tool for measuring willingness to care for older adults among undergraduate nursing students (King et al., 2013; Rathnayake et al., 2016).

Numerous factors can influence nursing graduates’ willingness to care for older people as their future professional practice, such as attitude towards and perception of older people care (Che et al., 2018; Haron et al., 2013; Zhang et al., 2016). The length of time spent with older adults is also positively related to the level of willingness to care for older people among nursing students (Chi et al., 2016). However, Rathnayake et al., (2016) reported that nursing students show little willingness to care for older people despite having a moderately positive attitude towards the practice. Nursing students’ willingness to care for older people is not considerably affected by their grades and whether they are living with their grandparents or studying gerontology courses (Chi et al., 2016). Age and gender also do not significantly influence nursing students’ willingness to care for older people (Carlson & Idvall, 2015). Therefore, the predictors of undergraduate nursing students’ willingness to care for older people remain inconclusive.

The healthcare system in Hong Kong and Mainland China has a severe shortage of nursing human resources (Chan et al., 2013; Wu et al., 2016). The healthcare system in Mainland China differs considerably from that in Hong Kong (Kong et al., 2015). Most healthcare services are provided by public hospitals in Mainland China, but private health services are also widely available in Hong Kong (Kong et al., 2015). However, nursing students in both regions are influenced by traditional Chinese culture, such as Confucianism (i.e. belief in filial piety and hierarchy in power, authority, and position; Lam, 2015). Consequently, nursing students believe that “older people should always be respected because of the hierarchy of elder‐younger” based on moral standards rooted in Confucianism (Lam, 2015, p. 46). Therefore, comparing willingness to care for older people between undergraduate nursing students in Hong Kong and those in Mainland China would be interesting, as the two regions have similar cultures but different healthcare systems.

The quality and quantity of nursing care for older people are highly dependent on the willingness and preparedness of future nurses (Hanson, 2014; Rababa et al., 2020). However, little is known about the willingness and attitude of undergraduate nursing students in Hong Kong and Mainland China towards older adult care and the factors associated with this practice. The findings of this study may provide relevant information for the development of a nursing curriculum and nursing education policies for enhancing older people care in the future.

This study aims to investigate willingness to care for older people among undergraduate nursing students in Hong Kong and Mainland China. This study specifically assesses (a) the students’ willingness to care for older people and (b) the predictors influencing the nursing students’ willingness to care for older people. The following four research questions are asked:

1. What is the level of willingness to care for older people of undergraduate nursing students in Hong Kong and Mainland China?
2. What is the relationship between demographic characteristics and willingness to care for older people among undergraduate nursing students in Hong Kong and Mainland China?
3. What is the relationship between social characteristics and willingness to care for older people among undergraduate nursing students in Hong Kong and Mainland China?
4. What is the relationship between attitude and perception of elderly care and willingness to care for older people among undergraduate nursing students in Hong Kong and Mainland China?

Three null and alternative hypotheses were proposed in this study:

Null hypotheses:

1. Willingness to care for older people is not associated with demographic characteristics among undergraduate nursing students in Hong Kong and Mainland China.
2. Willingness to care for older people is not associated with social characteristics among undergraduate nursing students in Hong Kong and Mainland China.
3. Willingness to care for older people is not associated with attitude and perception of elderly care among undergraduate nursing students in Hong Kong and Mainland China.

Alternative hypotheses:

1. Willingness to care for older people is associated with demographic characteristics among undergraduate nursing students in Hong Kong and Mainland China.
2. Willingness to care for older people is associated with social characteristics among undergraduate nursing students in Hong Kong and Mainland China.
3. Willingness to care for older people is associated with attitude and perception of elderly care among undergraduate nursing students in Hong Kong and Mainland China.

## 3 METHOD

### 3.1 Design

A correlational and cross‐sectional design was used in this study.

### 3.2 Setting, sample, and sample size

A total of three universities in Hong Kong (*n* = 1) and Mainland China (*n* = 2), where the researchers are presently affiliated, were chosen as the setting of this study. Convenience sampling was used, and undergraduate nursing students in Hong Kong and Mainland China were invited to participate in the study. The inclusion criterion was that the students must be currently enrolled in the undergraduate nursing program in their second, third, or fourth year. Nursing students in their first year were excluded, as they lacked major nursing courses and clinical experience or contact with patients.

The sample size was estimated via power analysis. Although the correlation coefficient between attitude towards ageing and willingness to care for older people ranged from 0.252 to 0.342 (Che et al., 2018; Rathnayake et al., 2016), no significant relationship was observed between attitude towards ageing and willingness to care for older people as the students’ first choice (Rathnayake et al., 2016). Hence, a highly conservative approach was used to set the effect size to 0.1. Coupled with a power of 0.80 and an alpha of 0.05 for the two‐tailed tests, the estimated total sample size was 783 (Portney & Watkins, 2009).

### 3.3 Ethical considerations

Ethical approval was obtained from the Human Research Ethics Committee of the local university (Ref: HSEARS20180530002). Information was provided to the undergraduate nursing students during the recruitment stage, including their voluntary participation and the benefits, risks, and significance of the study. The students were given sufficient time to ask questions. The students were also given the freedom to participate in the study. Moreover, the researchers had no knowledge of which of the students answered the questionnaire, as they did not have a list of the names of the students. The researchers ensured confidentiality and the students’ anonymity throughout the research period. No personal information was recorded, and informed consent was obtained from each respondent. For the online survey, the respondents’ consent to participate in the study was implied by their completion of the survey.

### 3.4 Measures

#### 3.4.1 *Sociodemographic* *data*

Part one of the survey collected information on the respondents’ age, gender, academic year level, family structure (nuclear or extended family), residential community type (rural or urban), and parents’ ages and whether they were living with their grandparent(s) and their perceived closeness to their grandparent(s) (on a visual analogue scale ranging from 0 [not close] to 10 [very close]).

#### 3.4.2 *Attitude* *towards ageing*

The respondents’ attitude towards ageing was measured with Kogan's Attitudes Toward Older People (KAOP) scale (Kogan, 1961). The KAOP scale includes 34 items (including 17 positive and 17 negative items) and uses a six‐point Likert‐type scale ranging from 1 (strongly disagree) to 6 (strongly agree). Total KAOP scale scores range from 34 to 204, with high scores representing a positive attitude. This scale is widely used for assessing nursing students’ attitude towards ageing (Faronbi et al., 2017; King et al., 2013; Rathnayake et al., 2016). The researchers translated the KAOP scale into Chinese, and the Cronbach's alpha value of the Chinese version of the KAOP scale was 0.75. Several studies support the satisfactory reliability and validity of this scale (Faronbi et al., 2017; Lambrinou et al., 2009; Yen et al., 2009).

#### 3.4.3 *Perception* *of older people care*

The respondents’ perception of older people care was measured with the Perspectives on Caring for Older Adults (PCOP) scale. The PCOP scale consists of 24 items and uses a five‐point Likert‐type scale ranging from 0 (strongly disagree) to 4 (strongly agree). The total score was obtained by adding up the individual responses. However, 12 negatively worded items must be scored in reverse before the summation. PCOP scale scores range from 0 to 96, with high scores indicating a positive perception of older people care. This scale was recently used in studies examining the perception of nursing students of caring for older people (Burbank et al., 2018; Redfield et al., 2016). The PCOP scale was translated into Chinese, and its reliability and validity were recently established, with satisfactory results. The Cronbach's alpha value of the Chinese version of the PCOP scale was 0.77, and the construct validity of the six‐factor structure was established using exploratory factor analysis (Cheng et al., 2020).

#### 3.4.4 *Willingness* *to care for older people*

Part four of the questionnaire focused on willingness to care for older people, which was measured with an item adopted from previous studies (King et al., 2013; Rathnayake et al., 2016). This item required the respondents to rank their preference to work with different age groups. The seven age group categories included newborns, infants (birth to 1 year), children (2 years–12 years), adolescents (13 years–18 years), young adults (19 years–39 years), middle‐aged adults (40 years–59 years), and older people (60 years and above). Prior research explored students’ preference order across nine areas of clinical practice, thereby confirming various populations as a reliable and valid measure for nursing students’ patient population preference (Briscoe, 2004; Happell, 2002; King et al., 2013; Rathnayake et al., 2016). Willingness to care for older people is measured with a single rated item rather than a multidimensional scale; hence, internal reliability was not reported.

The item required the respondents to “rank” rather than “rate” the seven categories. This format prompted the respondents to compare each category and list the order of their preference. The answers can sincerely reflect the number of respondents who preferred to work with older people compared with the other age groups. The students’ first choice age group and choice ranking for the older people age group (score: first choice = 1 and seventh choice = 7) were calculated. Low‐ranking scores indicated a high willingness to care for older people.

### 3.5 Data collection

Data collection was conducted from September 2018 to November 2018 using two approaches, namely, an onsite completion survey and an online survey. All eligible nursing students from three universities in Hong Kong (*n* = 1) and Mainland China (*n* = 2) were invited to participate in the survey. The eligible students who agreed to participate in the study were asked to give their implied/verbal consent (i.e. implicitly or verbally granted through a person's actions, such as responding to the questionnaire), which is a typical method for online surveys and questionnaires without a record of identity (Cruz et al., 2018; Lam et al., 2018; Suen et al., 2019). (a) *Onsite completion survey:* after providing consent, the students were given the four‐part self‐administered questionnaire, with a blank white envelope. The respondents were instructed on how to answer the questionnaire and requested to place the answered questionnaire in the designated envelope, seal the envelope, and return it to the researchers. The researchers then stored the envelopes containing the questionnaires in a locked cabinet until data collection was completed. (b) *Online survey:* an invitation letter to participate in the study, including the link to the online questionnaire, was sent to the fourth‐year nursing students who were allocated to different nursing placement hospitals in China through Wenjuanxing, which is a popular Chinese professional survey website (https://www.wjx.cn/). The online survey was developed interactively and pilot‐tested by the authors.

### 3.6 Statistical analyses

The data analyses were conducted using SPSS version 22.0. Descriptive statistics were used for the demographic characteristics of the respondents. T‐tests were conducted to examine the differences in demographics, willingness to care for older people, attitude towards ageing, and perception of older people care among the nursing students in Hong Kong and Mainland China. The significant difference in attitude towards ageing, perception of older people care, and work preference for and selection of caring for older people as a future career between the nursing students in Hong Kong and those in Mainland China was calculated as an effect size (Cohen, 1992). Multiple linear regression was performed on willingness to care for older people, with a hierarchical block design for the regression analysis, to determine the separate contributions of demographics, social characteristics, attitude towards ageing, and perception of older people care. First, the respondents’ age and gender were included in the hierarchical linear regression. Second, the respondents’ social characteristics were added, as reflected by their years of study, family structure, parents’ ages, and residential community type and whether they were living with their grandparents and their closeness to their grandparents. Third, the respondents’ attitude towards ageing and perception of older people care were added. The level of significance for all the statistical analyses was set to *p* ≤ .05. The initial statistical analyses were conducted separately, as the healthcare system and nursing education in Mainland China and Hong Kong differ considerably. With this understanding, the inferential statistics were used appropriately to indicate the salient predictors of the Chinese nursing students’ willingness to care for older people collectively owing to their similar cultural background.

## 4 RESULTS

Table 1 presents the sociodemographic data of the participants. The total sample consisted of 820 undergraduate nursing students, with 481 students (481/560, response rate = 85.9%) in Hong Kong and 339 students (339/386, response rate = 87.8%) in Mainland China. The mean age of the participants was 20.41 years (*SD* ± 1.35), ranging from 17 years to 34 years (Table 1).

**TABLE 1:** Respondents’ characteristics

	All (*N* = 820)	Hong Kong (*N* = 481)	Mainland China (*N* = 339)
Participant age; Mean (*SD*)	20.41 ± 1.35	20.29 ± 1.48	20.57 ± 1.15
Participant gender; No. (%)			
Male	145 (18.0%)	104 (22.32%)	41 (12.13%)
Female	659 (82.0%)	362 (77.68%)	297 (87.87%)
Level of BSN program; No. (%)			
2nd year	230 (25.2%)	153 (32.42%)	77 (22.78%)
3rd year	313 (34.2%)	161 (34.11%)	152 (44.97%)
4th year	267 (29.2%)	158 (33.47%)	109 (32.25%)
Structure of family; No. (%)			
Nuclear family	569 (70.77%)	391 (83.91%)	178 (52.66%)
Extend family	235 (29.23%)	75 (16.10%)	160 (47.34%)
Participant's father age; Mean(*SD*)	50.99 ± 6.94	54.70 ± 6.48	46.18 ± 3.92
Participant's mother age; Mean(*SD*)	48.17 ± 4.97	49.32 ± 5.22	46.51 ± 4.07
Living with grandparents			
No	576 (74.42%)	390 (87.84%)	186 (55.69%)
Yes	198 (25.58%)	54 (12.16%)	148 (44.31%)
Closeness with grandparents	6.23 ± 2.67	5.53 ± 2.39	7.33 ± 2.60
Type of community			
Rural area	254 (31.99%)	32 (7.00%)	222 (66.07%)
Urban area	540 (68.01%)	426 (93.00%)	114 (33.93%)

Abbreviation: BSN, Bachelor of Science in Nursing.

### 4.1 Attitude towards ageing, perception of older people care, and willingness to care for older people

The mean scores of attitude towards ageing of the participants in Hong Kong and those in Mainland China were 121.39 (*SD* = 11.93) and 115.05 (*SD* = 12.20), respectively. The mean scores of perception of older people care of the participants in Hong Kong and those in Mainland China were 74.80 (*SD* = 7.42) and 71.57 (*SD* = 5.87), respectively. These two variables showed the significant differences between the nursing students in Hong Kong and those in Mainland China, and the effect size of the difference in these two variables was “medium” in degree (Cohen, 1992; Table 2). The scores of willingness to care for older people were similar in the two groups (mean score = 4.44 versus. 4.68), and no significant difference was observed between the students in the two regions (*p* =.071).

**TABLE 2:** Attitudes toward aging, older people care perception and willingness to care for older people

Outcomes	Hong Kong, (*N* = 481)	Mainland China, (*N* = 339)	*t*	*p* value	*d*
	(Mean ± *SD*)	(Mean ± *SD*)			
Attitudes toward aging (KAOP) (range = 24–192)	121.39 ± 11.93	115.05 ± 12.20	7.39	<.001**	0.53
Elderly care perception (PCOP) (range = 49–107)	74.80 ± 7.42	71.57 ± 5.87	6.65	<.001**	0.48
Willingness to care for older people (range = 1–7)	4.68 ± 1.88	4.44 ± 1.60	1.80	.071	

Note

An independent sample *t*‐test was performed to assess whether there was difference between the two groups.

Abbreviations: KAOP, Kogan's Attitudes toward Older People Scale; PCOP, Perspectives on Caring for Older Adults Scale PCOP.

** Significant at the 0.01 level (2‐tailed).

### 4.2 Work preference

The majority of the participants in Hong Kong preferred to work with young adults (28.9%), followed by newborns (19.0%) and adolescents (18.3%). Only 7.7% of the nursing students in Hong Kong ranked caring for older people as their first choice of work (Table 3 and Table 4). “Work preference” for patient age stratum was tested and showed a significant difference between the nursing students in Hong Kong and those in Mainland China (χ^2^ = 21.64; *df* = 6; *p* =.001). Table 3 presents the significant difference, with a small effect size, in “work preference” for “older people” and “newborns.” Meanwhile, the majority of the participants in Mainland China preferred to work with young adults (34.6%), followed by adolescents (23.0%) and children (11.0%). Only 3.6% of the nursing students in Mainland China ranked caring for older people as their first choice of work (Table 3 and Table 4). The difference in caring for “older people” as their first choice of work between the nursing students in Hong Kong and those in Mainland China was significant (χ^2^ = 30.70; *df* = 6; *p* <.001). The significant difference, with a small effect size, in the selection of caring for older people as the “first choice of work” and “seventh choice of work” between the nursing students in Hong Kong and those in Mainland China is reported in Table 4.

**TABLE 3:** Work preference

	Hong Kong, (*N* = 467)		Mainland China, (*N* = 334)		χ^2^; *p* value	Effect size (phi‐coefficient)
	Number	Percentage	Number	Percentage		
New born	86	19.0	32	9.5	12.10; .001**	0.12
Infants	46	10.2	31	9.3		
Children	53	11.7	37	11.0		
Adolescents	83	18.3	77	23.0		
Young adults	131	28.9	116	34.6		
Middle age adults	33	7.3	29	8.7		
Older people	35	7.7	12	3.6	5.37; .021*	0.08
χ^2^	21.64					
*p* value	.001					

* Significant at the 0.05 level.

** Significant at the 0.01 level.

**TABLE 4:** Ranks distribution in selection of caring older people as a future career choice

	Hong Kong, (*N* = 453)		Mainland China, (*N* = 324)		χ^2^; *p* value	Effect size (phi‐coefficient)
	Number	Percentage	Number	Percentage		
First choice	35	7.7%	12	3.6%	5.38; .02*	0.08
Second choice	27	6.0%	21	6.3%		
Third choice	60	13.2%	50	14.9%		
Fourth choice	91	20.1%	91	27.2%		
Fifth choice	70	15.5%	61	18.2%		
Sixth choice	55	12.1%	50	14.9%		
Seventh choice	115	25.4%	39	11.6%	21.18; <.001**	0.17
χ^2^	30.70					
*p* value	<.001					

* Significant at the 0.05 level.

** Significant at the 0.01 level.

### 4.3 Predictors of willingness to care for older people

Tables 5, 6, and 7 present the results of the hierarchical multiple linear regression for willingness to care for older people. According to model 3 in Table 7, null hypothesis 1 was accepted, and null hypotheses 2 and 3 were rejected, while alternative hypothesis 1 was rejected, and alternative hypotheses 2 and 3 were accepted. The final model (model 3) was considered as the best competing model according to the increasing Nagelkerke *R* ^2^ values. For the nursing students in Hong Kong, the final model (Model 3) showed that undergraduate nursing program year 2 (*p* < .05) and perception of older people care (*p* < .05) were positively and significantly associated with willingness to care for older people, whereas age (*p* < .05) was negatively and significantly associated with willingness to care for older people (Table 5). The young nursing students in Hong Kong with a high perception of older people care were likely to have a high willingness to care for them. No salient predictors of willingness to care for older people were observed in the nursing students in Mainland China (Table 6). Among the nursing students in Hong Kong and Mainland China, the final model (Model 3) showed that undergraduate nursing program year 2 (*p* < .05) and perception of older people care (*p* < .05) were positively and significantly associated with willingness to care for older people (Table 7). Thus, the young nursing students with a high perception of older people care were likely to have a high willingness to care for them.

**TABLE 5:** Hierarchical regression modeling of predictors of willingness to care for the older people (Hong Kong, *N* = 481)

	Model 1 β’s (95% CI)	Model 2 β’s (95% CI)	Model 3 β’s (95% CI)
Demographic characteristics			
Age	−0.32 (−0.45, −0.19)**	−0.20 (−0.36, −0.04)*	−0.18 (−0.34,−0.02)*
Gender (Female)	−0.25 (−0.73, 0.24)	−0.19 (−0.68, 0.30)	−0.17 (−0.66, 0.33)
Social characteristics			
Level of BSN program			
2nd year		0.74 (0.18, 1.31)*	0.68 (0.12, 1.24)*
3rd year		0.33 (−0.19, 0.84)	0.24 (−0.32, 0.72)
4th year		1	1
Structure of family		0.03 (−0.64, 0.70)	0.04 (−0.62, 0.71)
Participant's father age		0.02 (−0.07, 0.02)	0.01 (−0.07, 0.02)
Participant's mother age		−0.03 (−0.02, 0.05)	−0.02 (−0.02, 0.05)
Living with grandparents		0.04 (−0.75, 0.82)	−0.04 (−0.82, 0.74)
Closeness with grandparents		−0.01 (−0.10, 0.07)	−0.01(−0.09, 0.08)
Type of community		−0.04 (−0.85, 0.78)	−0.08 (−0.89, 0.73)
Attitude and perception of elderly care			
Attitudes toward aging (KAOP)			−0.00 (−0.02, 0.02)
Elderly care perception (PCOP)			0.04 (0.01, 0.07)*
*R* ^2^	0.07	0.09	0.11

Note

BSN, Bachelor of Science in Nursing; KAOP, Kogan's Attitudes toward Older People Scale; PCOP, Perspectives on Caring for Older Adults Scale PCOP.

* Significant at the 0.05 level (2‐tailed).

** Significant at the 0.01 level (2‐tailed).

**TABLE 6:** Hierarchical regression modeling of predictors of willingness to care for older people (Mainland China, *N* = 339)

	Model 1 β’s (95% CI)	Model 2 β’s (95% CI)	Model 3 β’s (95% CI)
Demographic characteristics			
Age	−0.31 (−0.18, 0.13)	0.07 (−0.14, 0.29)	0.05 (−0.16,0.26)
Gender (Female)	0.16 (−0.39, 0.70)	0.17 (−0.38, 0.73)	0.24 (−0.31, 0.80)
Social characteristics			
Level of BSN program			
2nd year		0.48 (−0.28, 1.24)	0.41 (−0.35, 1.17)
3rd year		0.07 (−0.58, 0.72)	−0.02 (−0.67, 0.63)
4th year		1	1
Structure of family		0.30 (−0.09, 0.68)	0.28 (−0.11, 0.66)
Participant's father age		−0.01 (−0.07, 0.04)	−0.01(−0.08, 0.05)
Participant's mother age		−0.01 (−0.08, 0.05)	−0.02 (−0.07, 0.05)
Living with grandparents		0.06 (−0.30, 0.43)	0.34 (−0.33, 0.40)
Closeness with grandparents		−0.04 (−0.12, 0.03)	−0.04 (−0.16, 0.03)
Type of community		−0.24 (−0.63, 0.16)	−0.27 (−0.66, 0.13)
Attitude and perception of elderly care			
Attitudes toward aging (KAOP)			0.01 (−0.00, 0.03)
Elderly care perception (PCOP)			0.02 (−0.01, 0.06)
*R* ^2^	.001	.035	.057

Note

BSN, Bachelor of Science in Nursing.

Significant at the 0.05 level (2‐tailed).

Significant at the 0.01 level (2‐tailed).

**TABLE 7:** Hierarchical regression modeling of predictors of willingness to care for older people (Hong Kong and Mainland China, *N* = 820)

	Model 1 β’s (95% CI)	Model 2 β’s (95% CI)	Model 3 β’s (95% CI)
Demographic characteristics			
Age	−0.17 (−0.32, 0.13)**	−0.09 (−0.24, 0.06)	−0.09 (−0.24,0.01)
Gender (Female)	−0.53 (−0.46, 0.26)	−0.01 (−0.41, 0.32)	0.00 (−0.36, 0.37)
Social characteristics			
Location (Hong Kong)		0.12 (−0.01, 0.86)	0.06 (0.21, 0.68)
Level of BSN program			
2nd year		0.15 (0.17, 1.02)**	0.13 (0.10, 0.94)*
3rd year		0.05 (−0.17, 0.57)	0.02 (−0.29, 0.45)
4th year		1	1
Structure of family		0.04 (−0.15, 0.48)	0.04 (−0.16, 0.48)
Participant's father age		0.03 (−0.02, 0.04)	0.04 (−0.02, 0.04)
Participant's mother age		−0.06 (−0.06, 0.01)	−0.06 (−0.06, 0.01)
Living with grandparents		0.06 (−0.09, 0.44)	0.04 (−0.15, 0.39)
Closeness with grandparents		−0.03 (−0.08, 0.03)	−0.03 (−0.08, 0.03)
Type of community		−0.09 (−0.68, 0.04)	−0.09 (−0.68, 0.04)
Attitude and perception of elderly care			
Attitudes toward aging (KAOP)			0.03 (−0.01, 0.02)
Elderly care perception (PCOP)			0.13 (0.01, 0.06)**
*R* ^2^	.029	.055	.075

Note

BSN, Bachelor of Science in Nursing.

* Significant at the 0.05 level (2‐tailed).

** Significant at the 0.01 level (2‐tailed).

## 5 DISCUSSION

This study investigated willingness to care for older people among undergraduate nursing students in Hong Kong and Mainland China by considering major factors, that is, their demographics, social characteristics, attitude towards ageing, and perception of older people care. Based on the descriptive statistics, the results indicated that a very low proportion of the undergraduate nursing students in Hong Kong (7.7%) and Mainland China (3.6%) ranked caring for older people as their first choice of work, which received the lowest score among all the age groups (i.e. newborns, infants, children, adolescents, and young, middle‐aged, and older adults; Table 3). The difference in “work preference” for “older people” and “newborns” between the nursing students in Hong Kong and those in Mainland China was significant. In terms of the first, second, and seventh choice rankings for caring for older people as a future career, the results in Table 4 indicate that caring for older people was consistently less preferred by the nursing students in Hong Kong and Mainland China. Therefore, the promotion of positive experiences and interactions between nursing students and older people should be included in undergraduate nursing programs.

These results of this study are similar to those of a study on nursing students in Sri Lanka (5.1%), who chose caring for older people as their first choice (Rathnayake et al., 2016). Henderson et al., (2008) reported that most nursing students do not want to work with older people after graduation. A replicated longitudinal survey claimed that only three out of 150 nursing graduates want to work with older people (Stevens, 2011). Global healthcare market employers generally respect the work preference of nurses owing to the persistent problem of nurse shortages (Wang & Geraghty, 2017). The results of this study can raise a consistent global alarm for the need of the potentially insufficient workforce to care for the current and future ageing society.

Insufficient evidence exists on whether attitude towards ageing and perception of older people care affect willingness to care for older people among healthcare professionals owing to inconsistent research results (Che et al., 2018; Haron et al., 2013; Rathnayake et al., 2016; Zhang et al., 2016). A negative attitude towards caring for older people was observed among undergraduate nursing students (Celik et al., 2010; Henderson et al., 2008). A study conducted in Mainland China reported that willingness to care for older people among nursing students is at a medium‐to‐high level (the mean score was 3.55 on a five‐point Likert scale; Zhang et al., 2016). Moreover, social characteristics were inconclusive as predictors of willingness to care for older people. A study in Sri Lanka indicated that nursing students’ experience of living with older relatives has a positive influence on the development of a positive attitude towards older people. Living with older relatives can provide nursing students with a different perspective (Rathnayake et al., 2016). However, according to another study, living with older relatives does not significantly affect nursing students’ attitude towards older people (Hweidi & Al‐Obeisat, 2006).

These inconsistent findings failed to provide sufficient evidence on the salient predictors of willingness to care for older people among nursing students. The current study adopted a rigorous statistical method to delineate the potential predictors (i.e. demographics, social characteristics, attitude towards ageing, and perception of older people care) of willingness to care for older people in a large sample size. The results of model 3 (Tables 5 and 6) indicated that though several significant predictors were identified from the Hong Kong sample, the factors mentioned above could not predict willingness to care for older people, considering the low explained variance (*R* ^2^ = 5.7% to 11%) of the model. Such a result is important and striking, as modifying attitude towards ageing or perception of older adult care seems helpful for improving nursing students’ willingness to care for older people as their future work. A large sample size involving other variables and settings or qualitative inquiry may help in uncovering potential predictors.

The results of this study also indicated that significant differences existed in demographics, attitude towards ageing, and perception of older people care among the nursing students in Hong Kong and Mainland China. The students’ family structures and whether they were living with their grandparents and their closeness to their grandparents differed. The participants in Hong Kong had higher scores in attitude towards ageing and perception of older people care than those in Mainland China (*t* = 6.65–7.39, *p* <.001), and a “medium” effect size was observed in the difference between the two groups in attitude towards ageing and perception of older people care (Cohen, 1992). However, no significant difference was found in willingness to care for older people (*t* = 1.8, *p* =.071) between the participants in Hong Kong and those in Mainland China. These findings echoed the current regression analyses and revealed that demographics, attitude towards ageing, and perception of older people care may not be associated with future practice (i.e. willingness to care for older people). However, the participants in Mainland China showed slightly more willingness to care for older people than those in Hong Kong; thus, future studies can try to determine the reasons behind the career choices of nursing students in Mainland China, which can help in developing gerontological nursing education and practices geared towards enhancing older people care.

### 5.1 Limitations

The main limitation of this study is the limited number of universities in Hong Kong and Mainland China from which the respondents were selected. Although the sampled universities offered standard nursing programs with accreditation from corresponding nursing councils, the quality of their students compared with those of other universities in Hong Kong and Mainland China is uncertain. Therefore, the results may not be generalized to all undergraduate nursing students in Hong Kong and Mainland China. The design of this study was quantitative, thereby limiting the deep and thorough understanding of the reasons behind the nursing students’ career choices and low willingness to care for older people. This study considered all possible factors, but no salient predictors of willingness to care for older people could be identified. Thus, a qualitative inquiry may help in uncovering potential predictors.

## 6 CONCLUSIONS

The results of this study indicated that a very low proportion of the undergraduate nursing students, most of whom had a negative attitude towards ageing, ranked caring for older people as their first choice of work. Thus, nursing schools should adopt different educational strategies focussing on developing undergraduate nursing students’ willingness to care for older people. A “medium” effect size was observed in the difference in attitude towards ageing and perception of older people care between the nursing students in Hong Kong and those in Mainland China. This study failed to provide sufficient evidence on the salient predictors of this willingness among the undergraduate nursing students in Hong Kong and Mainland China. Therefore, further qualitative studies are needed to gain a deep understanding of the reasons behind undergraduate nursing students’ career choices.

## CONFLICT OF INTEREST

The authors declare that they have no competing interests.

## AUTHOR CONTRIBUTIONS

Study design: L.X.L., S.C.L. and H.L.C.; Data collection: H.L.C and X.L.L.; Data analysis: L.X.L., S.C.L. and H.L.C.; Manuscript preparation: L.X.L., S.C.L. and H.L.C.

## Data Availability

The data that support the findings of this study are available on request from the corresponding author. The data are not publicly available due to privacy or ethical restrictions.
